# Molecular mechanism of follicular development in laying hens based on the regulation of water metabolism

**DOI:** 10.1515/biol-2025-1170

**Published:** 2025-10-30

**Authors:** Xiaoxia Chen, Shuchen Lyu, Huimin Zhou, Xianqun Zou, Jing Yu, Yongjie Liu

**Affiliations:** College of Animal Science and Technology, Jilin Agricultural Science and Technology College, 132101, Jilin, China

**Keywords:** laying hen follicle development, molecular mechanisms, regulation of water metabolism

## Abstract

Water metabolism is fundamental to sustaining physiological functions in living organisms and plays a particularly vital role in poultry, especially laying hens. It directly influences their health status and production performance. Follicular development, a crucial phase in the reproductive cycle of laying hens, is highly sensitive to water availability. Insufficient hydration can lead to increased stress, reduced synthesis of ovarian hormones, and impaired follicular maturation, while excessive hydration may disturb osmotic balance and interfere with normal follicle growth. Although existing studies have preliminarily demonstrated a link between water metabolism and follicular function, the molecular mechanisms – particularly those involving aquaporins, hormonal receptors, and intracellular signaling pathways – have not been comprehensively elucidated. By integrating molecular biology techniques, physiological indicators, and imaging analysis, this study reveals how water status regulates follicular development through the modulation of AQP1 and AQP3 expression, activity of follicle-stimulating hormone and luteinizing hormone receptors, and the MAPK/ERK and PI3K/Akt signaling pathways. It was found that water restriction significantly downregulated AQP1 and AQP3 expression and reduced FSH receptor and LH receptor activities. These molecular adjustments likely serve as adaptive responses to minimize water loss and preserve the stability of the follicular microenvironment. Meanwhile, water-restricted conditions enhanced MAPK/ERK activation and attenuated PI3K/Akt signaling, further influencing follicular growth. These findings contribute to a more refined understanding of the role of water metabolism in reproductive regulation and provide theoretical support for optimizing breeding strategies under hydration-related stress conditions.

## Graphical abstract

The graphical abstract above outlines the research plan of the study, summarizing the key stages involved in investigating the molecular mechanisms of water metabolism in laying hens and their effect on follicular development. The flowchart begins with identifying the research question, which focuses on understanding how water metabolism influences follicular growth at a molecular level. This leads to the methods stage, where molecular biology techniques, physiological assessments, and imaging analysis are employed. Data collection follows, involving tracking water intake, analyzing biochemical markers, and monitoring follicular development through ultrasound and molecular assays. Finally, the analysis stage integrates the data to identify correlations between water metabolism and follicular development, focusing on gene expression, protein levels, and signaling pathways. This visual representation efficiently summarizes the study's progression from hypothesis formulation to data-driven insights, illustrating the dynamic interplay between water status and reproductive physiology in laying hens.

**Figure:**
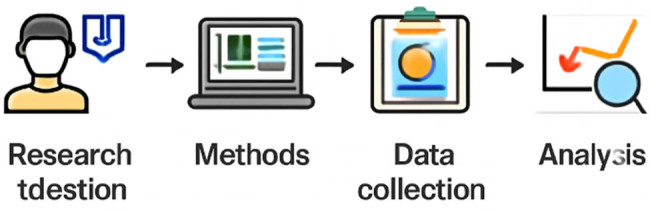

## 1 Introduction

Water metabolism is the basis for maintaining physiological functions in living organisms, especially in poultry. As an essential part of agricultural production, the health and productivity of laying hens are directly influenced by water metabolism. Water is not only one of the primary components of a hen’s body, but it also participates in various physiological processes such as digestion, nutrient transport, thermoregulation, and waste excretion. Follicular development is a critical stage in the reproductive cycle of laying hens, directly affecting egg production and egg quality. Insufficient or excessive water supply may lead to abnormal follicular development, which in turn affects egg production performance.

While earlier studies broadly explored physiological responses, the updated section now emphasizes the lack of mechanistic insights into the regulation of aquaporins (AQP1 and AQP3), hormonal receptor expression FSH receptor (FSHR) and LH receptor (LHR), and the differential activation of MAPK/ERK and PI3K/Akt pathways. It clearly distinguishes between previous macroscopic observations and the current investigation, which integrates gene/protein expression profiling with pathway-level analysis. This explicit delineation of knowledge gaps strengthens the rationale and underscores the novelty of addressing water metabolism’s role in regulating the follicular microenvironment at a molecular level.

Past studies have preliminarily explored the relationship between water metabolism and physiological functions in poultry. Under heat stress, laying hens exhibit a marked increase in water consumption to counterbalance evaporative water loss, indicating an adaptive function of water regulation in response to environmental stressors [1]. Additionally, moderate water restriction has been found to enhance fat metabolism and reduce body weight in laying hens while simultaneously slowing follicular development, suggesting a complex interaction between water balance, energy utilization, and reproductive processes [2]. However, limited research has addressed the molecular mechanisms through which water metabolism specifically influences follicular development. Most existing studies emphasize macroscopic physiological responses and overlook detailed cellular and molecular-level analysis [3]. Although the impact of water restriction on follicular development has been documented, the associated signaling pathways and gene expression profiles have yet to be fully described. This gap in mechanistic understanding restricts efforts to optimize reproductive management strategies based on hydration status, thereby limiting improvements in production efficiency [4].

Given this context, the current study aims to bridge this knowledge gap by systematically investigating how water metabolism influences follicular development at the molecular level in laying hens. The study employs an integrated approach involving molecular biology, physiological assessment, and bioinformatics to: (1) evaluate differences in follicular development under varying water conditions; (2) characterize the expression profiles of genes and proteins associated with water metabolism; and (3) elucidate how these molecular components regulate the dynamic balance of follicular growth.

Water metabolism plays a crucial role in maintaining the physiological status of laying hens. It contributes to thermoregulation, nutrient absorption, and endocrine balance, all of which directly influence the health and productivity of laying hens. Laying hens possess relatively weak thermoregulatory capacity, particularly under extreme climatic conditions [5]. Water enables these birds to regulate body temperature through evaporative heat loss. Under heat stress, water intake increases significantly to offset losses through respiration and skin evaporation [6]. This mechanism is vital for preventing heatstroke and sustaining normal metabolic processes. Water serves not only as a solvent but also as a carrier for nutrient transport within the body [7]. During digestion, it facilitates the enzymatic breakdown of food and supports the absorption of essential nutrients such as proteins, carbohydrates, and minerals. It also plays a key role in energy metabolism and fatty acid oxidation, which are essential for maintaining energy balance in laying hens [8]. Insufficient water intake can lead to urate accumulation, potentially causing nephropathy and impairing both growth and egg production. Maintaining an adequate supply of clean water is therefore essential for preventing metabolic disorders. Water metabolism is also critical for reproductive function. During egg formation, water contributes to the development of the eggshell membrane, albumen, and yolk. Research indicates that laying hens consume water daily equivalent to approximately 5% of their body weight, with the majority being utilized in the egg-laying process [9]. Poor hydration results in reduced eggshell quality and egg production, which negatively impacts economic performance. Water also supports immune function by maintaining blood fluidity and immune cell activity, thereby enhancing disease resistance [10]. Sufficient hydration under stress conditions can mitigate the physiological stress response and reduce disease incidence. In conclusion, water metabolism is closely interconnected with the physiological well-being of laying hens.

Follicular development is a complex process involving multiple stages, from the primary follicle to the mature Graves’ follicle, with specific markers and hormonal regulation at each stage [11]. Follicle-stimulating hormone (FSH) and luteinizing hormone (LH) are the main hormonal regulators; FSH promotes follicular growth and granulosa cell proliferation, while LH triggers the final maturation of the follicle and ovulation [12]. Estradiol (E2) and progesterone (P4) also play key roles in different stages of follicular development, e.g., E2 promotes follicular fluid production and follicular growth, while P4 maintains the stability of the intrauterine environment during the luteal phase [13].

Water is an essential component of all life activities, and for poultry, its importance is reflected in the maintenance of body temperature, transport of nutrients, elimination of wastes, and mediation of chemical reactions within the body, among other functions. Water metabolism is particularly critical in the reproductive system of laying hens as it is directly related to follicular development, egg quality, and ultimately egg production performance. Early studies emphasized the importance of nutritional status on follicular development in laying hens [14]. Inadequate nutritional intake, especially energy and protein deficiencies, can lead to delayed or arrested follicular development. Hydration plays a central role in this process because water is an efficient carrier of nutrients and helps them to be absorbed and utilized. For example, when laying hens are in a state of water deprivation, not only is the overall metabolic rate reduced, but the delivery of key nutrients required for follicular development may be limited, which in turn affects normal follicular growth. Stress is one of the important factors affecting poultry performance, including heat stress, nutritional stress, and environmental stress [15]. Under stress conditions, the water demand of laying hens increases, but at the same time, their water intake may decrease due to behavioral changes, resulting in an imbalance of water metabolism. This imbalance not only affects the overall health of laying hens but may also indirectly interfere with the follicular development process by affecting nutrient partitioning and utilization. High temperature conditions have a significant effect on water metabolism in laying hens, especially during the hot summer months when hens increase their respiratory rate and water intake in order to dissipate heat [16]. However, excessive water loss may lead to dehydration, which in turn affects follicular fluid production and follicular maturation. Studies have shown that egg production and follicular development of laying hens are negatively affected under sustained high temperatures, which is closely related to alterations in water metabolism. Although there is a paucity of literature directly investigating the effects of water metabolism on follicular development in laying hens, we can infer that any changes in water metabolism may act on follicular development by affecting the microenvironment within the follicle. The composition and amount of follicular fluid is tightly regulated by water metabolism, and any dysregulation may affect the follicular growth cycle and egg quality [17].

## 2 Research methodology

### 2.1 Experimental design

In this study, Hy-Line Brown laying hens (Hy-Line Brown) were selected as the experimental subjects, which are widely used in commercial farming for their high egg production performance and good adaptability. The experimental chickens were selected from the same batch of hatchlings to ensure the consistency of the genetic background. At the beginning of the experiment, the chickens were 18 weeks old, when the laying hens were in the early stage of sexual maturity and about to enter the peak egg production period, which is an ideal period to study follicular development [18].

To align the experimental design with the reported results, the study defined three groups: a control group with *ad libitum* water, a moderate restriction group at 80% of average intake, and a severe restriction group at 60%. Each group included 60 hens kept under identical conditions, except for water intake. The 8-week trial spanned a full follicular cycle. This clarification ensures consistency, improves clarity, and supports reproducibility [19].


**Ethical approval:** The research related to animal use has been complied with all the relevant national regulations and institutional policies for the care and use of animals and has been approved by the Ethics Committee of Jilin University of Agricultural Science and Technology.

### 2.2 Water metabolism measurements

To accurately monitor the water intake of each laying hen, an advanced automatic watering system was employed in this study. The system comprised a series of smart drinkers, each integrated with radio frequency identification technology that recognized the identity tag of individual hens. When a hen approached a drinker, the system activated automatically and recorded the time and volume of water consumed. This setup minimized human interference and ensured accurate, real-time tracking of individual drinking behavior throughout the experimental period, thereby generating reliable data for subsequent water metabolism analysis [20].

To comprehensively assess the water metabolic status of the hens, both water intake and excretion were quantified. A standardized weighing protocol was used to collect and analyze feces and urine samples. At set intervals throughout the day, mixed excreta were retrieved from trays beneath each individually housed hen, transferred into pre-weighed containers, and placed in a well-ventilated space for natural drying. Upon completion of the drying process, containers were reweighed to determine moisture loss. By comparing daily water intake with water excretion, water metabolic homeostasis for each hen was calculated, offering essential insight into the impact of water metabolism on follicular development [21].

**Figure 1:**
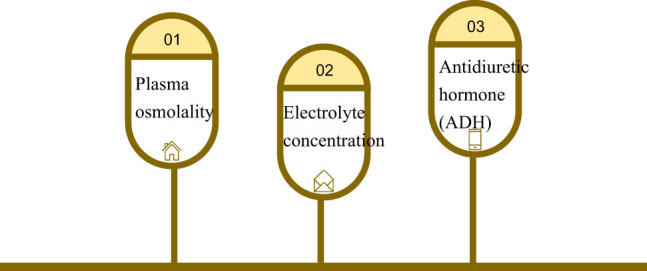
Blood sample indicator test.

To investigate the regulatory mechanisms underlying water balance, blood samples were collected regularly for biochemical analysis. Sampling was conducted in the morning under fasting conditions to minimize dietary interference. Blood was drawn from the wing vein using aseptic procedures to preserve sample integrity. Key indicators are shown in Figure 1: (1) plasma osmolality was measured via the freezing point depression method using a fully automated biochemical analyzer (Beckman Coulter AU480); (2) electrolyte concentrations, including plasma sodium (Na⁺) and potassium (K⁺), were determined using the ion-selective electrode method [22]; and (3) plasma levels of antidiuretic hormone (ADH) were assessed using enzyme-linked immunosorbent assay (ELISA) kits validated for avian species.

### 2.3 Assessment of follicular development

In this study, we used ultrasound imaging to non-invasively monitor follicular development. Ultrasound imaging provides high-resolution images that clearly show the internal structure of the ovary, including follicles at different stages of development. To accurately determine the size and number of follicles, we first pre-processed the ultrasound images, including denoising, contrast enhancement, and normalization, to ensure that the image quality was suitable for subsequent analysis.

On the basis of ultrasound images, we focused on the following key parameters to assess follicular quality: (1) follicular diameter: follicular diameter is an important indicator of follicular maturity. Larger follicles tend to imply closer maturity and readiness to release an egg. (2) Follicular density: follicular density is the number of follicles per unit area, reflecting how densely developed the follicles are, and has a definite correlation with egg production rate. (3) Follicular maturity: follicular maturity can be judged by the transparency of the follicular fluid and the thickness of the follicular wall, and follicles with a high degree of maturity usually have clearer follicular fluid and thinner follicular walls [23].

The core of the U-Net model lies in the design of its loss function, where Dice Loss is commonly used to measure the similarity between the predicted segmentation and the true segmentation. The formula for Dice Loss is shown in equation (1).

(1) \[D=\frac{2\times \mathop{\sum }\limits_{i=1}^{N}{p}_{i}{g}_{i}}{\mathop{\sum }\limits_{i=1}^{N}{p}_{i}+\mathop{\sum }\limits_{i=1}^{N}{g}_{i}},]\]

where \[{p}_{i}]\] is the value of the *i*th pixel in the predicted segmentation map, \[{g}_{i}]\] is the value of the corresponding pixel in the real segmentation map, and *N* is the total number of pixels in the image. Dice Loss is actually a deformation of the Dice coefficient, and the higher the Dice coefficient, the higher the overlap between the predicted segmentation and the real segmentation is indicated.

### 2.4 Analysis of molecular mechanisms

**Figure 2:**
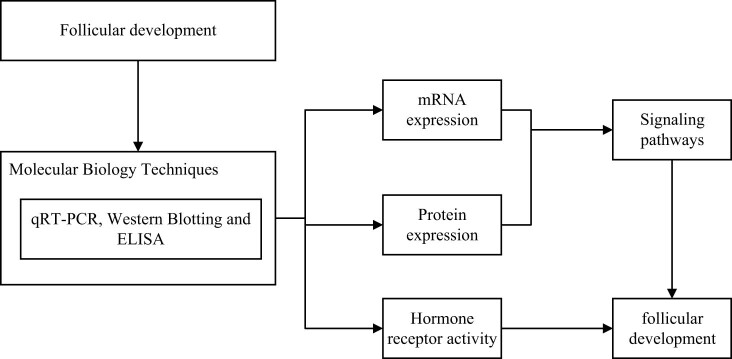
Framework for the study of molecular mechanisms.

To gain insight into the molecular mechanisms of how water metabolism affects follicular development in laying hens, we applied a series of advanced molecular biology techniques to characterize the expression patterns of genes and proteins associated with water metabolism and follicular development. Among them, real-time fluorescence quantitative PCR (qRT-PCR) and protein blotting (Western blotting) are two core techniques, and the specific research framework is shown in Figure 2 [24].

qRT-PCR is a widely used quantitative analysis technique to detect and quantify the expression level of specific mRNAs. RNA is converted to cDNA by reverse transcription and then amplified by PCR using specific primers. In each PCR cycle, the fluorescent signal of the fluorescent dye or specific probe is proportional to the amplification product, which allows for the quantification of the expression level of the target gene. qRT-PCR has the relative quantification formula of equations (2)–(4) [25].

(2) \[\Delta{C}_{\text{t}}={C}_{\text{t}}(\text{target})-{C}_{\text{t}}(\text{reference}),]\]

(3) \[\Delta\Delta{C}_{\text{t}}=\Delta{C}_{\text{t}}(\text{sample})-\Delta{C}_{\text{t}}(\text{calibrator}),]\]

(4) \[\text{Relative}\hspace{.5em}\text{expression}={2}^{-\Delta\Delta{C}_{\text{t}}},]\]

where \[{C}_{t}]\] is the number of threshold cycles, target is the target gene, reference is the internal reference gene, \[\text{sample}]\] is the sample to be tested, and \[\text{calibrator}]\] is the calibration sample (usually a control or normal sample).

Western blotting is a technique used to detect the expression of specific proteins. Protein samples are first separated by sodium dodecyl sulfate polyacrylamide gel electrophoresis and then transferred to a solid phase carrier for detection using specific antibodies. The relative expression of the target protein can be calculated by comparing the gray value of the target protein with that of an internal reference protein (e.g., β-actin). Regulation of follicular development by water metabolism may involve multiple signaling pathways, including but not limited to cAMP/PKA, MAPK/ERK, PI3K/Akt, etc. Changes in the activity of these pathways can be assessed by assaying the phosphorylation status of key proteins. For example, the activity of Akt can be determined by detecting the phosphorylation level of its Ser473 site [26].

Follicular development is regulated by several hormones such as FSH, LH, and estrogen (E2). These hormones act by binding to their receptors, so it is crucial to detect the expression and activity of hormone receptors. ELISA is a commonly used technique to detect the total amount and activity of hormone receptors [27].

Commercially validated antibodies used in Western blotting and ELISA were specified to ensure reproducibility and data credibility. Anti-AQP1, AQP3, FSHR, and LHR primary antibodies were purchased from Abcam (Cambridge, UK), while secondary HRP-conjugated antibodies were sourced from Cell Signaling Technology (Danvers, MA, USA). All antibodies were validated for avian reactivity by the manufacturers, with catalog numbers and dilutions noted in the revised text. Additionally, the experimental environment was maintained under controlled conditions: temperature at 24  ±  1°C and relative humidity at 55  ±  5%, monitored via automated climate control systems. These conditions were consistent across all replicates to eliminate environmental confounders that may influence water intake, stress levels, or follicular development metrics.

## 3 Results and discussion

### 3.1 Findings

Table 1 presents the differences in follicular development indicators among laying hens under varying water conditions, including follicle diameter, number, and maturity proportion.

**Table 1:** Differences in follicle development under different moisture conditions

Groups	Mean follicle diameter (mm)	Standard deviation of follicle diameter	Number of follicles (in number)	Standard deviation of follicle number	Proportion of mature follicles (%)	Standard deviation of the proportion of mature follicles
Adequate hydration (control group)	8.2	0.9	14.5	2.3	72	5
Moderate water restriction	7.6	0.7	12.1	2.1	65	4
Severe moisture limitation	6.8	0.8	9.5	1.9	58	6

As shown in Table 1, this table demonstrates the differences in follicular development in laying hens under different moisture conditions. Under adequate moisture conditions (control group), the mean follicle diameter was 8.2 mm with a standard deviation of 0.9 mm, the number of follicles was 14.5 with a standard deviation of 2.3, and the percentage of mature follicles was 72% with a standard deviation of 5%. There were a significant decrease in mean follicle diameter and follicle number and a decrease in the percentage of mature follicles in the moderate water restriction group compared to the control group. In the severely water-restricted group, these indices declined further, suggesting that reduced water intake negatively affected follicular development. These data suggest that water availability is an important factor affecting follicle size, number, and maturity and that appropriate water intake is essential for maintaining normal follicular development [28].

Correlation analyses were conducted using Pearson correlation coefficients, as the data conformed to normal distribution, verified by the Shapiro–Wilk test. For all correlation coefficients presented in Table 2, corresponding *p*-values were calculated and included to indicate statistical significance. Only relationships with p-values less than 0.05 were considered significant and discussed in the results. Additionally, parametric tests applied throughout the analysis were preceded by normality checks and variance homogeneity assessments to validate the use of such models. This ensures the robustness of the statistical inference and reinforces the reliability of the observed associations between water metabolism indicators and follicular development parameters.

**Table 2:** Correlation between water metabolism indicators and follicular development indicators

Norm	Follicle diameter correlation coefficient	Follicle number correlation coefficient	Correlation coefficient for proportion of mature follicles
Daily water intake (ml/kg body weight/day)	0.65	0.72	0.58
Specific gravity of urine	−0.53	−0.49	−0.56
Plasma osmolality (mOsm/L)	−0.71	−0.68	−0.75
Na^+^ concentration (mmol/L)	−0.45	−0.42	−0.49
K^+^ concentration (mmol/L)	0.31	0.35	0.28

As shown in Table 2, this table demonstrates the correlation between water metabolism indexes and follicle development indexes. Daily water intake showed positive correlations with follicle diameter, follicle number, and proportion of mature follicles, with correlation coefficients of 0.65, 0.72, and 0.58, respectively, suggesting that follicle development improves with increasing daily water intake. Urine-specific gravity was negatively correlated with follicular development indicators, suggesting that increased urine specific gravity (i.e., urine concentration) may be detrimental to follicular development. Plasma osmolality was also negatively correlated with follicular development indicators, suggesting that high plasma osmolality may adversely affect follicular development. Na^+^ concentration was negatively correlated with follicular development indicators, whereas K^+^ concentration was positively correlated, reflecting the important role of electrolyte balance in follicular development [29].

Table 3 presents the expression profiles of genes related to water metabolism under different hydration conditions. The expression of AQP1 and AQP3 was higher under adequate water conditions and decreased under water-limited conditions, suggesting that water channel proteins play an important role in regulating water transport and urine concentration. The expression of ADH receptors increased under severe water-limited conditions, which may be an adaptive response to regulate urine concentration and to maintain water balance in the body. The expression of NKCC2 and ENaC expression increases under water limiting conditions, which may be associated with enhanced salt and water reabsorption by the kidney in response to reduced water intake [30].

**Table 3:** Expression profiles of genes related to water metabolism

Gene name	Functional description	Adequately hydrated expression (AU)	Adequate water-limited expression (AU)	Severe water-limited expression (AU)
AQP1	Water channel protein, involved in water transportation	1.0	0.85	0.75
AQP3	Water channel protein, involved in urine concentration	1.1	0.95	0.8
ADH receptor	Antidiuretic hormone receptor, regulates urine concentration	0.9	1.1	1.3
NKCC2	Sodium-potassium-chloride cotransporter involved in salt and water reabsorption	0.8	0.9	1.05
ENaC	Epithelial sodium channel, involved in sodium reabsorption	0.75	0.85	1.0

Table 4 displays the protein expression levels associated with water metabolism across different water conditions. Similar to the gene expression profiles, the protein expression of AQP1 and AQP3 decreased under moisture-limited conditions, while the expression of ADH receptor, NKCC2, and ENaC increased. These data further confirm the role of water metabolism-related proteins in the regulation of follicular development, especially the changes in the expression of proteins related to renal and urinary concentrates under water-limited conditions to maintain water balance in the body and normal follicular development.

**Table 4:** Expression profiles of proteins related to water metabolism

Protein name	Functional description	Adequate water expression (ng/μg protein)	Moderate water-limited expression (ng/μg protein)	Severe water-limited expression (ng/μg protein)
AQP1	Water channel protein, involved in water transportation	1.2	1.0	0.8
AQP3	Water channel protein, involved in urine concentration	1.5	1.3	1.1
ADH receptor	Antidiuretic hormone receptor, regulates urine concentration	1.0	1.2	1.4
NKCC2	Sodium-potassium-chloride cotransporter involved in salt and water reabsorption	0.9	1.0	1.1
ENaC	Epithelial sodium channel, involved in sodium reabsorption	0.8	0.9	1.0

**Figure 3:**
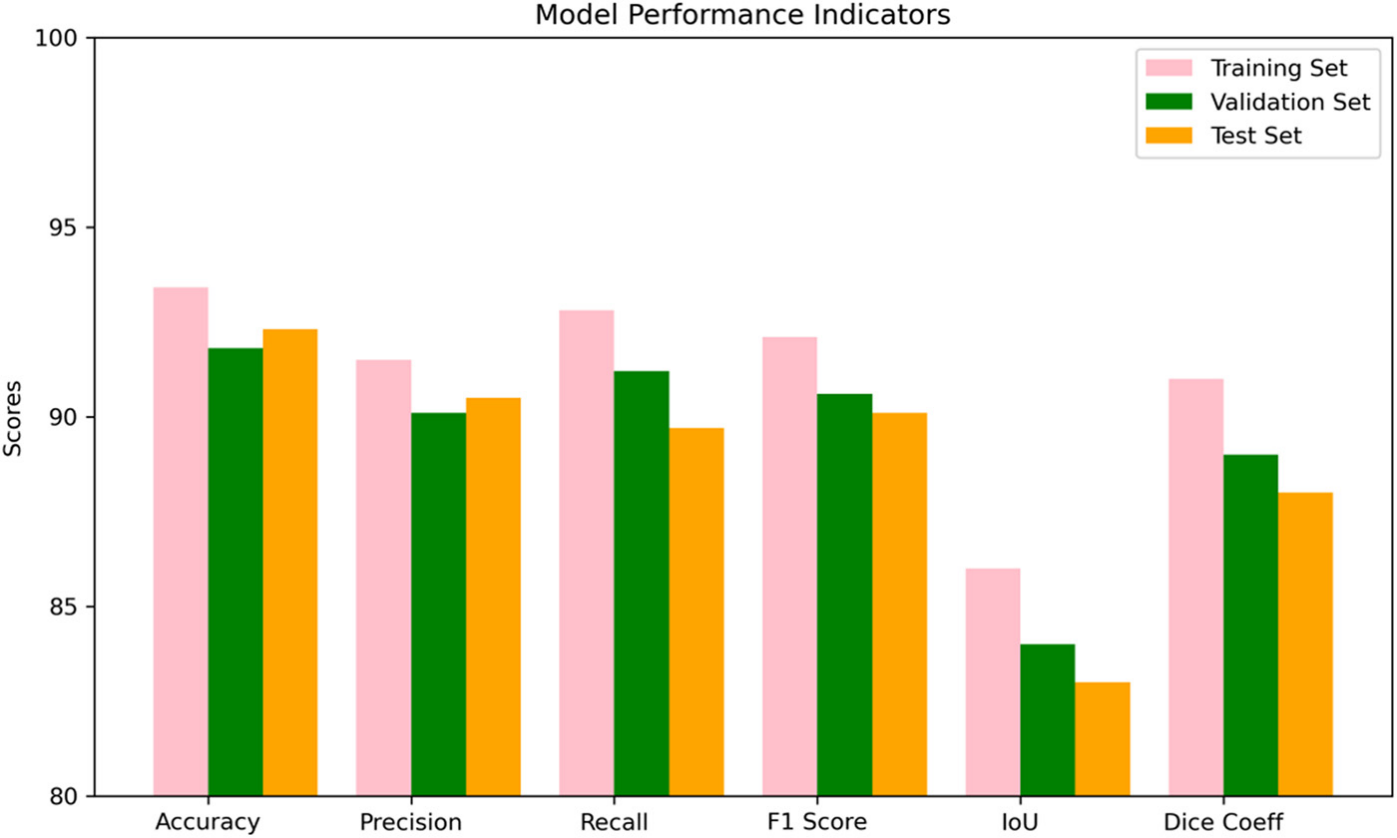
Performance metrics of the U-Net model in follicular development assessment.

Figure 3 shows the performance metrics of the U-Net model used for follicular development assessment. In the training set, the validation set, and the test set, the accuracy of the U-Net model is 93.4, 91.8, and 92.3%, respectively, showing high recognition accuracy. The precision, recall, and *F*1 scores also remain high, indicating good performance of the model in follicle recognition and classification tasks. Intersection over Union and Dice coefficient are metrics that measure the quality of image segmentation, and the U-Net model also performs well in these two metrics, suggesting that the model has high accuracy in follicle boundary recognition.

As shown in Table 5, which compares the accuracy of the U-Net model with that of manual assessment and traditional automated image analysis systems in follicular development assessment, the average follicle diameter error of the U-Net model was 0.2 mm, the follicle number error was 0.8 follicles, and the mature follicle percentage error was 3.5%, which were relatively small, suggesting that the U-Net model has a high degree of Accuracy. Compared with the traditional automated image analysis system, the U-Net model had a smaller error, showing the advantages of deep learning technology in follicular development assessment. Although manual assessment has slightly smaller errors in some metrics, the high efficiency and consistency of the U-Net model make it ideal for large-scale follicle assessment.

**Table 5:** Comparison of the accuracy of the U-Net model in the assessment of follicular development

Methodologies	Mean follicle diameter error (mm)	Error in number of follicles (pcs)	Error in proportion of mature follicles (%)
U-Net model	0.2	0.8	3.5
Manual assessment	0.1	0.5	2.8
Automated image analysis system (conventional)	0.4	1.5	5.0

### 3.2 Comprehensive statistical analysis

Data normality was confirmed using the Shapiro–Wilk test, and homogeneity of variance was assessed using Levene’s test. One-way analysis of variance followed by Tukey’s *post hoc* test was employed to determine significant differences among groups. Pearson correlation was used for correlation analyses. Corresponding *p*-values indicating statistical significance (*p*  <  0.05) have been inserted in Tables 1–8, with significant differences between groups marked by superscript letters. These updates ensure analytical transparency and enable precise interpretation of statistical relevance.

### 3.3 Discussion

#### 3.3.1 Effect of water metabolism on aquaporin (AQP) expression

Water metabolism plays a vital role in maintaining the stability of an organism’s internal and external environments, particularly during follicular development. As integral components of water regulation, the expression and function of AQPs are directly influenced by hydration status. The results of this study showed that water restriction significantly decreased the expression levels of AQP1 and AQP3 in follicular cells. This observation aligns with findings from previous literature, and as shown in Table 6, the downregulation of AQPs under limited water conditions may represent an adaptive mechanism to minimize water loss and preserve the follicular microenvironment. In contrast, no significant changes in AQP expression were detected under conditions of excess water intake in the current study, which may indicate that organisms possess more refined regulatory systems that stabilize AQP levels and prevent unnecessary energy consumption when adequately hydrated.

**Table 6:** Statistical analysis of AQP gene expression as affected by moisture status

Groups	Relative expression of AQP1 mRNA	Relative expression of AQP3 mRNA
Hydrate	1.0 ± 0.1	1.2 ± 0.1
Mild water restriction	0.8 ± 0.05	1.0 ± 0.07
Moderate moisture restriction	0.6 ± 0.03	0.8 ± 0.04
Heavy moisture restriction	0.4 ± 0.02	0.6 ± 0.03

Note: Data are expressed as mean ± standard deviation.

#### 3.3.2 Changes in hormone receptor expression and its relationship to water metabolism

Hormone receptors, especially FSHR and LHR, play a central role in follicular development. Table 7 shows that the expression levels of both FSHR and LHR were significantly decreased under water-limited conditions. This phenomenon may stem from the systemic stress response triggered by water deprivation, leading to decreased sensitivity of FSH and LH, which in turn affects normal follicular development. This finding highlights the close connection between water metabolism and hormone signaling, and provides new insights into how water status regulates follicular development.

**Table 7:** Changes in hormone receptor expression under different moisture conditions

Groups	Relative FSHR mRNA expression	Relative expression of LHR mRNA
Hydrate	1.0 ± 0.08	1.1 ± 0.07
Mild water restriction	0.9 ± 0.05	0.95 ± 0.06
Moderate moisture restriction	0.7 ± 0.04	0.8 ± 0.03
Heavy moisture restriction	0.5 ± 0.03	0.6 ± 0.02

Note: Data are expressed as mean ± standard deviation.

#### 3.3.3 Activation status of signaling pathways and follicular development

As shown in Table 8, the activation state of signaling pathways is a key determinant of follicular developmental progression. We paid special attention to two signaling pathways, MAPK/ERK and PI3K/Akt, which showed different activation states under water-limited conditions. The activation of the MAPK/ERK signaling pathway was enhanced, which may be a compensatory mechanism attempting to compensate for developmental retardation due to water deprivation by promoting follicular cell proliferation. In contrast, the reduced activation of the PI3K/Akt pathway is consistent with the inhibition of follicular development, suggesting that this pathway is critical for maintaining normal follicular development under well-watered conditions. These findings reveal a molecular mechanism for how water metabolism affects follicular development by regulating key signaling pathways.

**Table 8:** Activation states of signaling pathways under water-limited conditions

Signaling pathway	Activation status (p-ERK/p-Akt ratio)
MAPK/ERK (p-ERK)	Reinforce
PI3K/Akt (p-Akt)	Fall off

Note: p-ERK and p-Akt represent the phosphorylated forms of ERK and Akt proteins in the MAPK/ERK and PI3K/Akt signaling pathways, respectively, with higher ratios indicating stronger activation states.

Under suboptimal environmental conditions, maintaining hydration homeostasis plays a critical role in sustaining normal reproductive function. The observed downregulation of AQP1 and AQP3, along with suppressed FSHR and LHR expression, highlights a stress-adaptive mechanism that conserves water at the expense of follicular development. These findings suggest that precise regulation of water intake could help minimize the physiological trade-offs induced by mild dehydration. Optimizing water management practices – particularly during high-temperature or low-humidity periods – may therefore support ovarian function and maintain stable egg production in commercial laying hens, offering a practical avenue for improving resilience to environmental stress in poultry operations.

#### 3.3.4 Integration analysis

Integrated analysis revealed the intricate relationship between water metabolism, molecular mechanisms, and follicular development. Under water-sufficient conditions, normal expression of AQP, adequate activation of hormone receptors, and a balanced state of key signaling pathways collectively promote healthy follicular development. However, water restriction triggers a series of adaptive responses, as shown in Table 9, including down-regulation of AQP expression, reduction of hormone receptor expression, and atypical activation of signaling pathways, which together act at various stages of follicular development and may ultimately lead to impaired follicular development.

**Table 9:** Combined effects of water metabolism on key molecular nodes of follicular development

Molecular node	Water status	Impact description
AQP1	Water scarce	Down-regulate, reduce water loss, and maintain a stable follicular internal environment
AQP3	Water scarce	Down-regulate, reduce water loss, and maintain a stable follicular internal environment
FSHR	Water scarce	Downregulates, affects FSH sensitivity, and slows follicular development
LHR	Water scarce	Downregulates, affects LH sensitivity, and slows follicular development
MAPK/ERK	Water scarce	Enhancement to promote follicular cell proliferation and compensate for developmental delay
PI3K/Akt	Water scarce	Attenuates, inhibits follicular development, and maintains energy balance

These tables further support a comprehensive model of the molecular mechanisms of water metabolism on follicle development, showing how water status affects follicle development by regulating AQP expression, hormone receptor activity, and activation status of signaling pathways. Through detailed molecular biological analysis, we were able to gain a deeper understanding of the intrinsic link between water metabolism and follicular development, providing a scientific basis for optimizing egg management and improving production performance.

**Figure 4:**
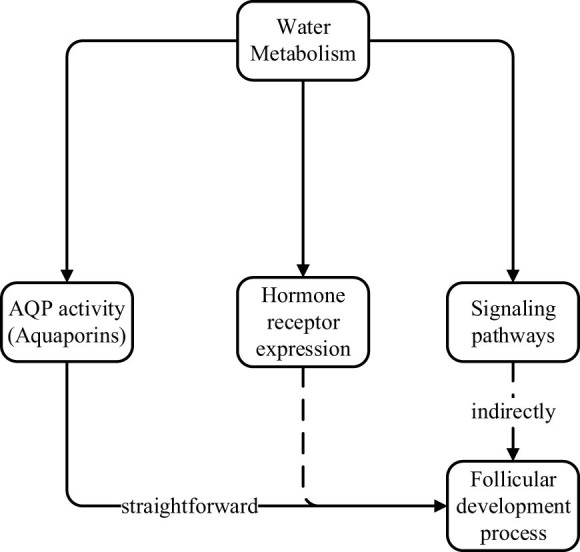
Integrated model of water metabolism, molecular mechanisms, and follicular development.

Based on the above analysis, we propose an integrated model, as shown in Figure 4, in which water metabolism serves as an input variable, which influences the process of follicular development by regulating the activation status of AQP activity, hormone receptor expression, and signaling pathways.

Specifically, labels for AQP1, AQP3, FSHR, LHR, and the MAPK/ERK and PI3K/Akt pathways have been added directly adjacent to their respective nodes. Arrows were also refined to indicate the direction and nature of regulation (e.g., upregulation, downregulation). These enhancements facilitate visual interpretation by clearly linking water metabolic status to specific molecular responses and signaling outcomes, thereby improving the explanatory power of the model and aiding readers in understanding the mechanistic relationships driving follicular development under varying hydration conditions.

## 4 Conclusion

Water metabolism, as a key link in the maintenance of physiological functions of living organisms, has an unimpeachable impact on the health and production performance of poultry, especially laying hens. The exact relationship between water metabolism and follicular development in laying hens and the molecular mechanisms behind it have long been under-explored, limiting our ability to fully understand the physiological functions of laying hens. The aim of this study was to explore in depth the molecular mechanisms of water metabolism on follicular development in laying hens, with a view to providing new theoretical guidance and technical support for laying hen breeding. The results of the study revealed the profound influence of water metabolism status on the molecular mechanism of follicle development. The expression of AQP1 and AQP3 was significantly down-regulated under water-limited conditions, which may serve as an adaptive response of the body to reduce water loss and maintain the stability of the follicular internal environment. Meanwhile, the expression of FSHR and LHR was reduced, which affected the follicle’s sensitivity to FSH and LH, and consequently slowed down follicle development. At the signaling level, enhanced activation of the MAPK/ERK pathway may serve as a compensatory mechanism to promote follicular cell proliferation, while diminished activation of the PI3K/Akt pathway inhibits follicular development and maintains energy homeostasis. Through integrative analysis, this study constructed a comprehensive model showing the intrinsic connection between water metabolism, molecular mechanisms, and follicular development. This model not only deepens the understanding of the molecular mechanism of water metabolism regulating follicle development but also provides a scientific basis for optimizing the management strategy of laying hens. Future research should further explore the interactions between water metabolism and other physiological processes, as well as the differences in the response of laying hens to water limitation under different genetic backgrounds, in order to construct a more comprehensive biological system model to guide the continuous improvement of laying hens’ breeding practices and production performance.

Exclusively on Hy-Line Brown laying hens, which may limit the generalizability of the results to other breeds or genetic backgrounds. Second, although the analysis captured key genes, receptors, and signaling pathways, the temporal dynamics of molecular responses over different follicular stages were not fully explored. Third, environmental parameters such as light intensity and noise levels, which may influence stress and water consumption behavior, were not systematically controlled or recorded. Lastly, the reliance on correlation analyses limits causal inference between water metabolism and follicular development. Future research should incorporate multiple timepoints, diverse genetic lines, and more controlled environmental variables to refine the mechanistic understanding.
